# Forensic Significance of Sternal Fusion in Adult Age Estimation: An Autopsy Study in North India

**DOI:** 10.7759/cureus.111263

**Published:** 2026-06-21

**Authors:** Shekher Pradeep, Hitesh Chawla

**Affiliations:** 1 Forensic Medicine & Toxicology, Shaheed Hasan Khan Mewati Government Medical College, Nalhar, IND

**Keywords:** age estimation, age-related variation, autopsy, forensic identification, sternum, xiphisternal joint, manubriosternal joint

## Abstract

Background: Age estimation is an important component of forensic identification, particularly in cases involving unknown, decomposed, mutilated, or skeletonized human remains. Following completion of skeletal maturity, commonly used indicators such as epiphyseal fusion become less reliable, making evaluation of other skeletal structures necessary. The sternum is considered useful in forensic age estimation because of its resistance to decomposition and progressive fusion changes during adult life. The present study was conducted to evaluate the relationship between age and fusion of different sternal elements in the North Indian population.

Materials and methods: A prospective cross-sectional study was conducted in a tertiary care centre of southern Haryana, India. A total of 115 medico-legal autopsy cases of known age, aged 15 years and above, were included in the study. The sternum was dissected during autopsy, and the fusion status of the body of the sternum, xiphisternal joint, and manubriosternal joint was assessed grossly. Statistical analysis was performed using IBM SPSS Statistics for Windows, version 29.0 (IBM Corp., Armonk, New York, United States), and associations between fusion patterns and age groups were evaluated using Pearson’s Chi-square test.

Results: Fusion of the body of the sternum was observed in almost all cases and did not show a statistically significant association with age (p = 0.910). In contrast, fusion of the xiphisternal joint and manubriosternal joint increased progressively with advancing age. Chi-square analysis demonstrated a statistically significant association between xiphisternal joint fusion and advancing age (χ² = 10.152, p = 0.038), while manubriosternal joint fusion approached statistical significance (χ² = 7.467, p = 0.058). Xiphisternal fusion was absent below 25 years and became progressively more frequent in older age groups, whereas manubriosternal fusion was predominantly observed after 35 years of age. Female cadavers demonstrated slightly earlier and higher fusion rates in advanced age groups compared to male cadavers. Binary logistic regression demonstrated that xiphisternal and manubriosternal fusion were significant independent predictors of age ≥45 years, with the combined model showing excellent discriminatory ability (area under the curve (AUC) = 0.896).

Conclusion: Fusions of the xiphisternal and manubriosternal joints are potential supportive indicators demonstrating significant age-associated trends, particularly beyond the fourth decade of life. Fusion of the body of the sternum has limited forensic significance because of its early completion and lack of progressive age-related variation. Assessment of sternal fusion should be interpreted in conjunction with other skeletal indicators and population-specific standards for improved medico-legal accuracy.

## Introduction

Age estimation is an important component of forensic identification. In medico-legal practice, identification becomes particularly challenging when dealing with mutilated, decomposed, burnt, dismembered, or skeletonized human remains where routine visual recognition is impossible. In children and adolescents, age estimation is relatively accurate due to predictable patterns of dental eruption and epiphyseal fusion of long bones [1]. However, after skeletal maturity, usually beyond 21-25 years of age, age estimation becomes increasingly difficult because skeletal growth is complete and assessment relies largely on degenerative and fusion-related changes in selected skeletal structures. Consequently, the margin of error in age estimation increases progressively with advancing age. Several skeletal structures have been studied for adult age estimation, including the pubic symphysis, auricular surface, cranial sutures, sternal end of ribs, clavicle, and sternum [2]. Among these, the sternum has attracted considerable forensic interest because of its central anatomical location, resistance to decomposition, and frequent recovery even in fragmented remains.

The sternum undergoes sequential developmental and degenerative changes throughout life, making it a potentially useful indicator for adult age estimation [2,3]. It develops from multiple ossification centers and demonstrates progressive fusion at different anatomical sites. Fusion of the sternebrae forming the body of the sternum generally occurs earlier in life, whereas fusion of the xiphisternal and manubriosternal joints tends to occur later and progresses with increasing age [3]. These fusion patterns have been investigated in different populations, and several studies have reported a significant relationship between sternal fusion and chronological age. However, considerable inter-individual and inter-population variations have also been observed. Factors such as genetics, nutritional status, socioeconomic conditions, physical activity, environmental influences, and overall health may influence the timing and progression of ossification and fusion.

Because of these variations, population-specific standards are essential for improving the accuracy and reliability of forensic age estimation. Although multiple studies on sternal fusion have been conducted in different regions of India and abroad, there remains a paucity of data from northern India, particularly southern Haryana. Regional studies are therefore necessary to establish locally relevant reference standards for medico-legal practice. Thus, the present study was undertaken to evaluate the age-related patterns of fusion of the body of the sternum, xiphisternal joint, and manubriosternal joint in a North Indian population and to assess the forensic utility of these fusion markers for adult age estimation.

## Materials and methods

This prospective cross-sectional study was conducted in the Department of Forensic Medicine at the Shaheed Hasan Khan Mewati Government Medical College, Nalhar, Haryana, India, between July 2024 and July 2025. The study was approved by the Institutional Ethics Committee of Shaheed Hasan Khan Mewati Government Medical College (protocol number: EC/OA-49/2024).

Study population

A total of 115 medico-legal autopsy cases aged 15 years and above with known age were included. Cases with fractured sternum, congenital or acquired sternal abnormalities, and unidentified bodies with unknown age were excluded.

Autopsy procedure

**Figure 1:**
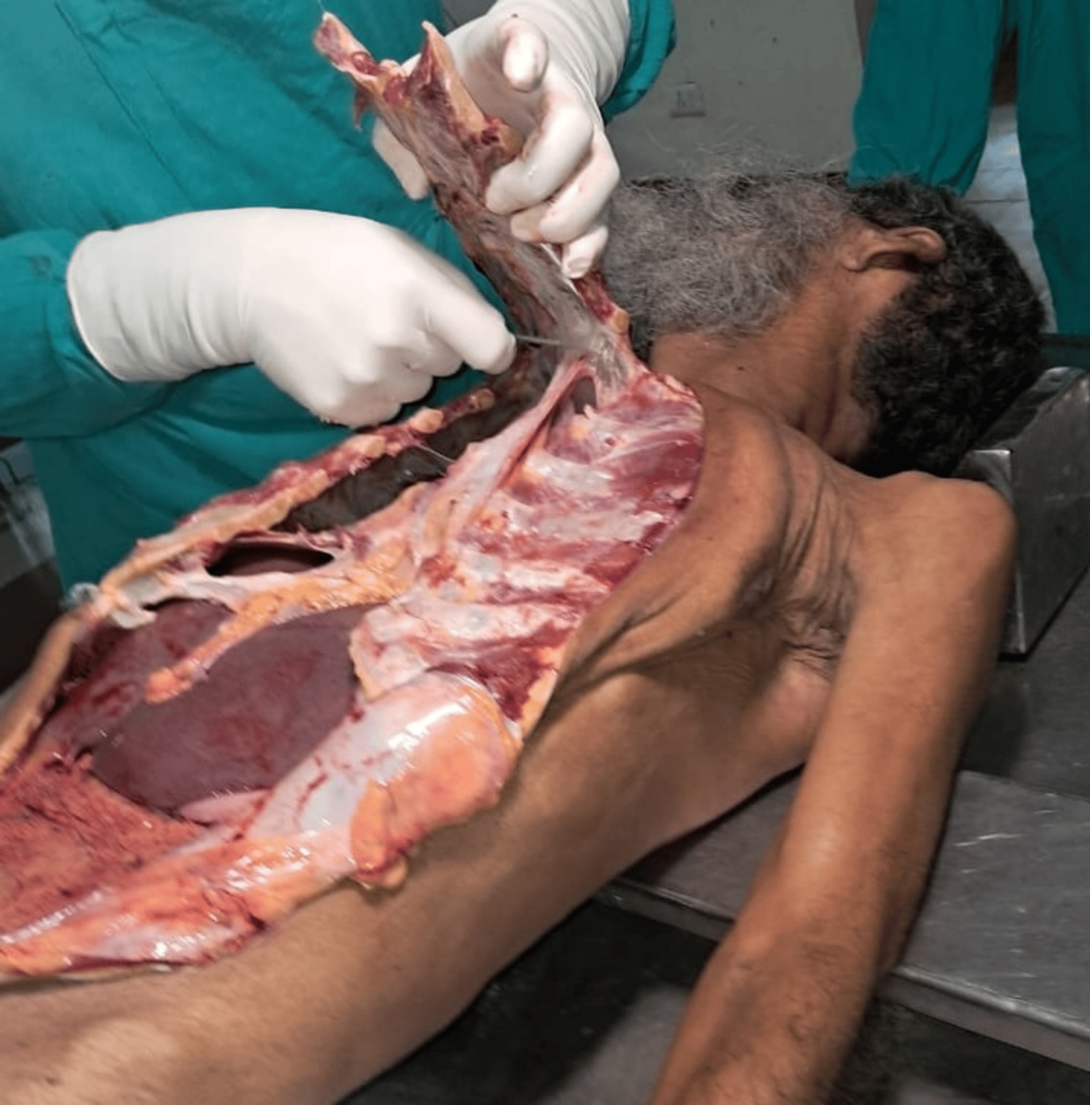
Removal of sternum during autopsy

During the autopsy, an I-shaped incision from the chin to the symphysis pubis was made to open the chest cavity. The skin, subcutaneous tissue, and muscles were separated. Ribs were dissected along the costochondral junction, and the intact sternum was removed after disarticulating the sternoclavicular joints (Figure 1). 

**Figure 2:**
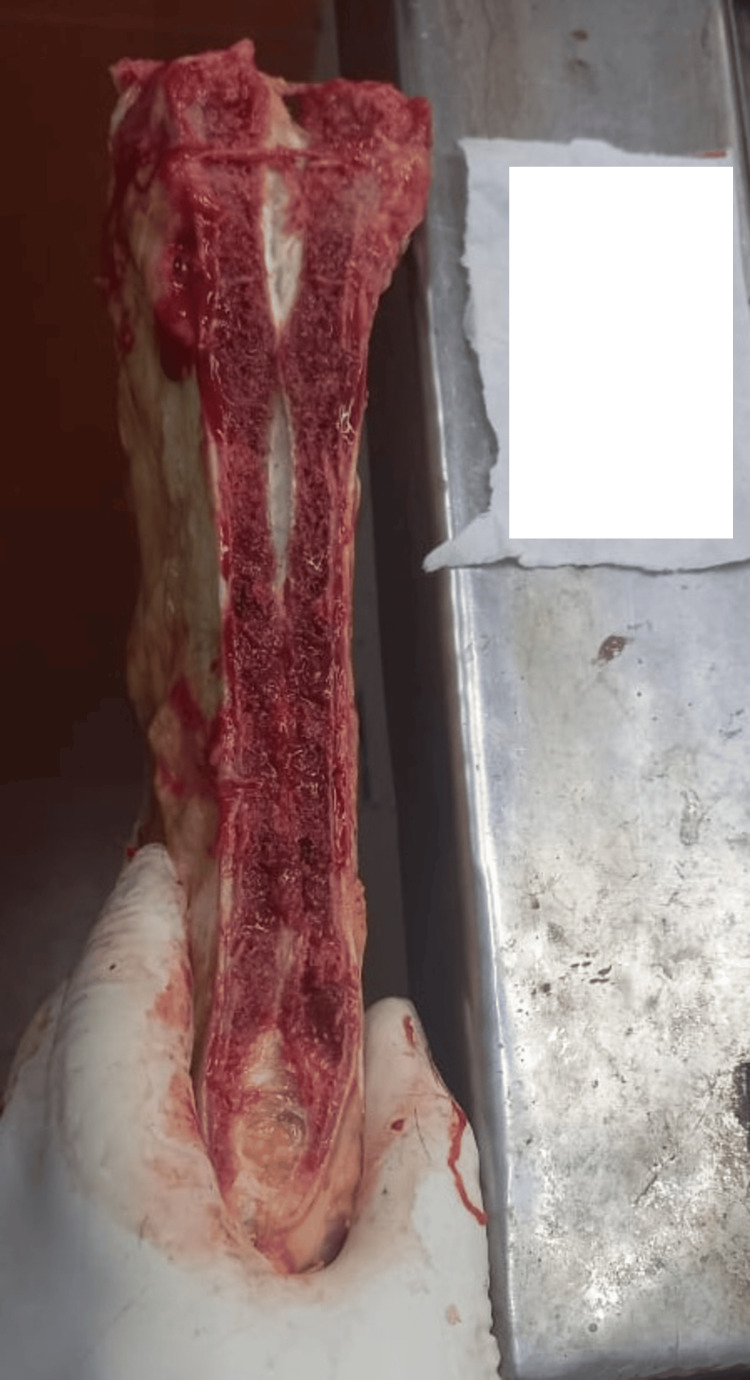
Gross specimen demonstrating fusion of body of sternum, xiphisternum, and manubriosternal joint

The soft tissues from the sternum were removed with the help of a scalpel and a brain knife. The sternum was then dissected vertically in the midline with an electric saw. Fusion was assessed by direct macroscopic examination. A joint was classified as fused when complete bony continuity was present without a visible joint space (Figure 2). A joint was classified as unfused when a distinct joint line remained visible. Partial fusion was not recorded separately. Full-thickness creamy white cartilage at the manubrium-sternal, first-second sternebrae, second-third sternebrae, and third-fourth sternebrae was taken to indicate no fusion. 

Serial sections of the xiphisternum from the tip to the body were made. If it cut smoothly, then it meant no fusion. If it cut with much resistance and grittiness, then it meant evidence of ossification. The findings at the manubrium-sternal, body of sternum, and xiphi-sternal part, whether fused or unfused, were correlated with the known age. To minimize the observer bias, all observations were made by the same observer. The observed findings were recorded in a pre-designed proforma and correlated with the known age of the deceased. 

Data analysis

The collected data were entered into Microsoft Excel 2016 (Microsoft Corporation, Redmond, Washington, United States) and analyzed using IBM SPSS Statistics for Windows, version 29.0 (IBM Corp., Armonk, New York, United States). Frequency and percentages were calculated for categorical variables, while mean and standard deviation (SD) were calculated for continuous variables. Pearson’s Chi-square test was applied to evaluate the association between fusion patterns and different age groups. Binary logistic regression analysis was performed to assess the predictive value of xiphisternal and manubriosternal fusion for age ≥45 years, and adjusted odds ratios (aORs) with 95% confidence intervals (CIs) were calculated. Diagnostic performance was evaluated by calculating sensitivity, specificity, positive predictive value (PPV), and negative predictive value (NPV). Receiver operating characteristic (ROC) curve analysis was performed to assess the discriminatory ability of the regression model, and the area under the curve (AUC) was determined. A p-value of less than 0.05 was considered statistically significant.

## Results

The study included 115 cadavers aged 15-90 years (mean age: 35.5 ± 16.3 years); 58.3% were male, while 41.7% were female. The majority of participants belonged to the 25-34-year age group. The distribution of study participants by age group and sex is represented in Table 1. The Chi-square test result (χ²=10.587, p-value 0.060) indicates that the difference in age group distribution between males and females was not statistically significant at the 0.05 level (Table 2).

**Table 1:** Distribution of study participants by age group and sex (N=115)

Age group (years)	Sex			
	Male (n=67)		Female (n=48)	
	Number	Percentage	Number	Percentage
15-24	12	17.91%	17	35.42%
25-34	24	35.82%	14	29.17%
35-44	17	25.37%	6	12.50%
45-54	5	7.46%	4	8.33%
55-64	6	8.96%	1	2.08%
65 and older	3	4.48%	6	12.50%

**Table 2:** Association between age group and sex among study participants: Pearson's Chi-square analysis

Chi-square	10.587
df	5
Sig.	0.060^a^

Table 3-8 summarizes the age-wise distribution of fusion patterns in the body of the sternum, xiphisternal joint, and manubriosternal joint among male and female subjects. Fusion of the body of the sternum was observed in nearly all individuals across all age groups and did not demonstrate a statistically significant association with advancing age (Tables 3, 4).

**Table 3:** Age- and sex-wise distribution of body of sternum fusion (N=115)

Age group (Years)	Body of sternum							
	Fused				Unfused			
	Sex				Sex			
	Male (n=66)		Female (n=47)		Male (n=1)		Female (n=1)	
	Frequency	Percentage	Frequency	Percentage	Frequency	Percentage	Frequency	Percentage
15-24	12	18.18%	16	34.04%	0	0.00%	1	100.00%
25-34	23	34.85%	14	29.79%	1	100.00%	0	0.00%
35-44	17	25.76%	6	12.77%	0	0.00%	0	0.00%
45-54	5	7.58%	4	8.51%	0	0.00%	0	0.00%
55-64	6	9.09%	1	2.13%	0	0.00%	0	0.00%
65 and older	3	4.55%	6	12.77%	0	0.00%	0	0.00%

**Table 4:** Association between body of sternum fusion and age group according to sex: Pearson's Chi-square analysis

	Fused	Unfused
Chi-square	9.786	2.000
Df	5	1
Sig.	0.082	0.157

Xiphisternal joint fusion was absent in the 15-24 year age group and first appeared predominantly in males aged 25-34 years, with fusion frequency increasing progressively thereafter (Table 5). Chi-square analysis demonstrated a statistically significant association between xiphisternal joint fusion and advancing age (χ² = 10.152, p = 0.038) (Table 6).

**Table 5:** Age- and sex-wise distribution of xiphisternal joint fusion

Age group (Years)	Xiphisternum							
	Fused				Unfused			
	Sex				Sex			
	Males (n=19)		Females (n=6)		Male (n=48)		Female (n=42)	
	Frequency	Percentage	Frequency	Percentage	Frequency	Percentage	Frequency	Percentage
15-24	0	0.00%	0	0.00%	12	25.00%	17	40.48%
25-34	1	5.26%	0	0.00%	23	47.92%	14	33.33%
35-44	5	26.32%	0	0.00%	12	25.00%	6	14.29%
45-54	5	26.32%	1	16.67%	0	0.00%	3	7.14%
55-64	5	26.32%	0	0.00%	1	2.08%	1	2.38%
65 and older	3	15.79%	5	83.33%	0	0.00%	1	2.38%

**Table 6:** Association between xiphisternum fusion and age group according to sex: Pearson's Chi-square analysis *:  Statistically significant at p < 0.05 level

	Fused	Unfused
Chi-square	10.152	8.690
Df	4	5
Sig.	0.038^*^	0.122

Manubriosternal joint fusion was absent in younger age groups and became increasingly evident after 35 years, particularly among female subjects in older age categories (Table 7). Manubriosternal joint fusion approached statistical significance (χ² = 7.467, p = 0.058) (Table 8). Overall, the combined assessment of xiphisternal joint and manubriosternal joint fusion patterns improved the forensic applicability of adult age estimation.

**Table 7:** Age- and sex-wise distribution of manubriosternal joint fusion

Age group (Years)	Manubriosternal joint							
	Fused				Unfused			
	Sex				Sex			
	Male (n=8)		Female (n=7)		Male (n=59)		Female (n=41)	
	Frequency	Percentage	Frequency	Percentage	Frequency	Percentage	Frequency	Percentage
15-24	0	0.00%	0	0.00%	12	20.34%	17	41.46%
25-34	0	0.00%	0	0.00%	24	40.68%	14	34.15%
35-44	1	12.50%	0	0.00%	16	27.12%	6	14.63%
45-54	0	0.00%	2	28.57%	5	8.47%	2	4.88%
55-64	4	50.00%	0	0.00%	2	3.39%	1	2.44%
65 and older	3	37.50%	5	71.43%	0	0.00%	1	2.44%

**Table 8:** Association between manubriosternal fusion and age group according to sex: Pearson's Chi-square analysis

	Fused	Unfused
Chi-square	7.467	7.667
Df	3	5
Sig.	0.058	0.176

Xiphisternal fusion showed 80.0% sensitivity and 91.1% specificity for predicting age ≥45 years. The PPV and NPV were 71.4% and 94.3%, respectively. Manubriosternal joint fusion demonstrated 56.0% sensitivity and 98.9% specificity for predicting age ≥45 years. The positive predictive value and negative predictive value were 93.3% and 89.0%, respectively. Binary logistic regression analysis demonstrated that both xiphisternal fusion and manubriosternal fusion were significant independent predictors of age ≥45 years. Presence of xiphisternal fusion increased the odds of belonging to the ≥45-year age group by 21-fold (aOR = 21.0, 95% CI: 5.25-84.0, p-value < 0.001), while manubriosternal fusion increased the odds by 42.3-fold (aOR = 42.3, 95% CI: 3.95-453.4, p = 0.002). The wide CIs observed in regression analysis likely reflect smaller sample sizes in older age groups. The borderline non-significant Chi-square association for manubriosternal fusion (p = 0.058) and the significant multivariable logistic regression result (p = 0.002) are not contradictory. Chi-square analysis represents an unadjusted bivariate comparison, whereas logistic regression estimates the independent contribution of fusion status after adjustment for covariates. Consequently, an association that is borderline in univariate analysis may become statistically significant in a multivariable model.

**Figure 3:**
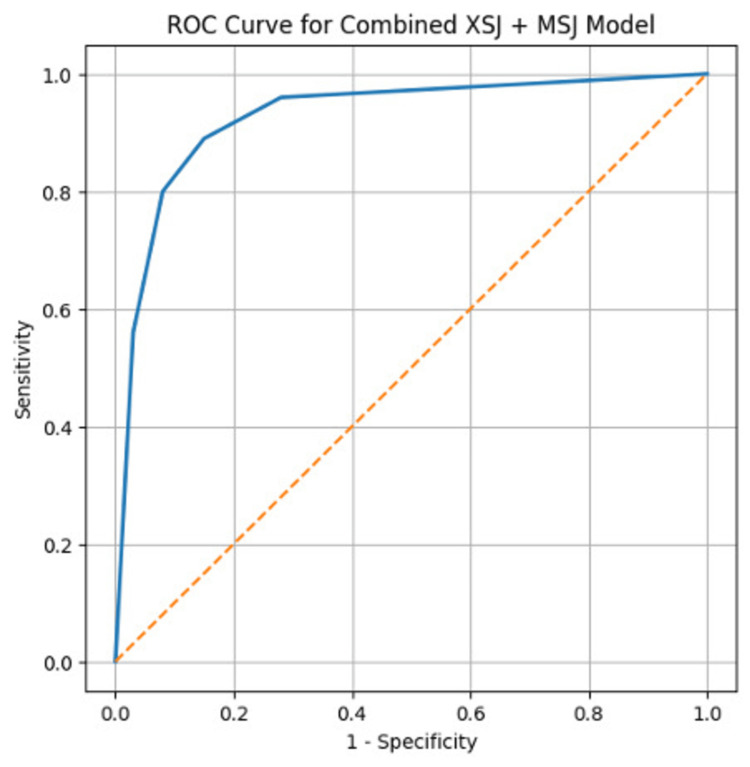
Receiver operating characteristic (ROC) curve demonstrating the predictive performance of the combined xiphisternal and manubriosternal fusion model for estimation of age ≥45 years. The blue curve represents the observed model performance, while the diagonal dotted line represents a non-informative test with no discriminatory ability (AUC = 0.5). AUC: area under the curve; XSJ: xiphisternal  joint; MSJ: manubriosternal joint

Although univariate Chi-square analysis for manubriosternal fusion approached statistical significance, it did not reach statistical significance (p = 0.058); multivariate logistic regression demonstrated significant predictive value after adjustment for xiphisternal fusion. ROC analysis demonstrated good discriminatory ability of the combined xiphisternal and manubriosternal fusion model for prediction of age ≥45 years (AUC = 0.896) (Figure 3).

## Discussion

The present study demonstrated that fusion of the body of the sternum occurs early in life and remains nearly universal throughout adulthood, thereby limiting its usefulness for adult age estimation. These findings are consistent with the observations of Nema et al. [4], Mukhopadhyay et al. [5], and Bacci et al. [6], who also reported no meaningful sex differences in sternebral fusion patterns. Although Manoharan et al. suggested that sternebral fusion may be useful irrespective of sex, their conclusions were largely based on younger age groups and smaller sample sizes [7]. The present study therefore reinforces the conclusion that fusion of the body of the sternum has negligible forensic value in adult age estimation.

In contrast, xiphisternal fusion showed a progressive increase with advancing age and demonstrated a significant association with chronological age. Female subjects in the present study demonstrated slightly earlier fusion, which may reflect earlier skeletal maturation; however, considerable overlap between sexes limits the reliability of sex-specific thresholds. Similar findings were reported by Chopra and Mahajan [8], Nagrale et al. [9], and Singh et al. [10], who attributed earlier fusion in female subjects to hormonal influences and accelerated skeletal maturation. However, studies by Badhe et al. [11] and Jain et al. [12] reported no statistically significant sex differences. 

Age- and sex-wise analysis of manubriosternal fusion demonstrated that female individuals showed relatively earlier and higher fusion rates in advanced age groups compared to male individuals. Similar trends were reported by Chopra and Mahajan [8] and Siddiqui et al. [13], whereas Jain et al. [12] and Ali et al. [14] found no significant sex differences. The borderline statistical significance observed in the present study may be due to the relatively smaller sample size in older age groups. Nevertheless, the findings support the concept that manubriosternal fusion increases progressively with advancing age and may occur slightly earlier in females. 

The comparative analysis presented in Table 9 demonstrates a consistent pattern across different populations and methodologies regarding the forensic utility of sternal fusion. Fusion of the body of the sternum occurs early in life and remains nearly universal throughout adulthood, thereby limiting its value for adult age estimation. In contrast, fusion of the xiphisternal and manubriosternal joints demonstrates a progressive relationship with advancing age and serves as a more reliable indicator in adult individuals. Nevertheless, significant inter-individual and inter-population variability exists, particularly in computed tomography-based and foreign population studies, indicating that sternal fusion should not be used as an isolated parameter for age estimation.

**Table 9:** Comparative analysis of fusion of sternal elements for age estimation in the literature

Author(s)	Year	Body of Sternum Fusion	Xiphisternal Joint Fusion	Manubriosternal Joint Fusion
Present study	2025	Fusion observed in almost all age groups; unfused cases extremely rare; no statistically significant association with age	Absent below 25 years; fusion increases progressively with age; significant age association, maximum fusion ≥65 years	Absent below 35 years; progressive increase with age; >50% fusion ≥65 years; significant association with age
Nema et al. [4]	2025	Lower body segments fused across most age groups; poor age correlation	Progressive fusion with age; variability in older age groups	Fusion increases with age but complete fusion seen even in younger adults
Chopra & Mahajan [8]	2024	—	Reliable mainly after 60–65 years	Fusion precedes xiphisternal joint; reliable after 50–60 years
Singh et al. [10]	2024	—	Mean fusion around 40 years; useful indicator in 4th decade	—
Siddiqui et al. [13]	2023	—	Complete fusion in all individuals ≥60 years	Complete fusion in ≥55–60 years
Sahu et al. [15]	2022	Useful mainly below 25 years	Begins after 30 years; supportive above 40 years	Wide overlap; partial fusion persists into old age
Mukhopadhyay et al. [5]	2021	Sternebrae fused by 17–20 years	Complete fusion may begin in early 30s but highly variable	Complete fusion seen early in some cases; absent even in elderly
Nagrale et al. [9]	2021	—	>90% fusion after 55–65 years	Complete fusion by 65–70 years
Bacci et al. [6]	2018	—	Fusion seen in both young and old adults; poor age predictability	Highly variable; unreliable as standalone marker
Jain et al. [12]	2018	—	—	Fusion seen as early as mid-20s; non-fusion persists into 70s
Manoharan et al. [7]	2016	Complete fusion of body segments by early adulthood	100% fusion after 60 years	Fusion begins late; incomplete fusion may persist
Silajiya et al. [16]	2013	—	Common after mid-40s	Partial fusion after 50 years; complete mainly after 59–64 years
Tayal et al. [17]	2013	Fusion of sternebrae complete by 25–30 years	Begins after 30 years; complete after 45–50 years	Begins after 40 years; invariably present after 55 years
Kaneriya et al. [3]	2013	Complete fusion by puberty to early adulthood	Almost invariably fused by 45–50 years	Consistent fusion after 55 years

The findings of the current study support the use of sternal fusion as a supportive adjunct rather than a definitive method for adult age estimation. Owing to biological variability, overlapping age ranges, and the relatively small sample size, sternal fusion should be interpreted in conjunction with other anthropological and forensic indicators.

Limitations of the study

Despite providing valuable insights into age estimation from the fusion of sternal elements, the present study has certain limitations. The relatively limited representation of older age groups may have reduced the precision of age-specific fusion estimates and contributed to wider confidence intervals in regression analysis. Fusion was categorized only as fused or unfused without grading partial fusion, potentially underestimating early fusion changes. Binary categorization of fusion status may have oversimplified transitional ossification changes and reduced the sensitivity of age estimation. Interobserver and intraobserver reliability analyses could not be performed because all observations were conducted by a single observer. Advanced imaging modalities such as computed tomography, which allow better visualization of partial fusion, were not employed. Selection bias may be present because the sample consisted exclusively of medico-legal autopsy cases and may not fully represent the general population. Furthermore, the small number of fused cases within certain age strata may have contributed to statistical instability and wide CIs for some regression estimates. Furthermore, the study evaluated sternal fusion largely in isolation rather than integrating multiple skeletal indicators into a predictive age estimation model. Future studies with larger sample sizes, computed tomography-based evaluation, and multifactorial models are recommended to improve the accuracy and reliability of adult age estimation.

## Conclusions

The different parts of the sternum can be useful in adult age estimation. Fusion of the body of sternum was observed in almost all individuals irrespective of age and therefore had limited value as an age indicator. In contrast, fusion of the xiphisternal and manubriosternal joints showed a gradual increase with advancing age and demonstrated a significant association with chronological age. Xiphisternal fusion appeared earlier and more consistently, while manubriosternal fusion was more commonly observed in older individuals.

The assessment of sternal fusion can serve as a supportive tool in forensic age estimation, particularly in adults where other skeletal indicators may be less reliable. However, sternal fusion should not be used alone and must be interpreted along with other skeletal and physiological parameters for better accuracy. The present study contributes useful reference data for this region population and highlights the continuing forensic relevance of sternal fusion in adult age estimation.
